# Potential Application of the Plant-Derived Essential Oils for Atherosclerosis Treatment: Molecular Mechanisms and Therapeutic Potential

**DOI:** 10.3390/molecules28155673

**Published:** 2023-07-26

**Authors:** Siarhei A. Dabravolski, Vasily N. Sukhorukov, Alexandra A. Melnichenko, Victoria A. Khotina, Alexander N. Orekhov

**Affiliations:** 1 Department of Biotechnology Engineering, Braude Academic College of Engineering, Snunit 51, P.O. Box 78, Karmiel 2161002, Israel; 2 Institute of General Pathology and Pathophysiology, 8 Baltiyskaya Street, 125315 Moscow, Russia; vnsukhorukov@gmail.com (V.N.S.); sasha.melnichenko@gmail.com (A.A.M.); nafany905@gmail.com (V.A.K.); a.h.opexob@gmail.com (A.N.O.)

**Keywords:** essential oils, atherosclerosis, inflammation, oxidative stress, endothelial dysfunction

## Abstract

Essential oils (EOs) are complex secondary metabolites identified in many plant species. Plant-derived EOs have been widely used in traditional medicine for centuries for their health-beneficial effects. Some EOs and their active ingredients have been reported to improve the cardiovascular system, in particular to provide an anti-atherosclerotic effect. The objective of this review is to highlight the recent research investigating the anti-inflammatory, anti-oxidative and lipid-lowering properties of plant-derived EOs and discuss their mechanisms of action. Also, recent clinical trials exploring anti-inflammatory and anti-oxidative activities of EOs are discussed. Future research on EOs has the potential to identify new bioactive compounds and invent new effective agents for treatment of atherosclerosis and related diseases such as diabetes, metabolic syndrome and obesity.

## 1. Introduction

Atherosclerosis is an advanced chronic inflammatory disease, characterised by a pathological remodelling of the arterial wall, lipid accumulation and build-up of atheromatous plaque. Advanced atherosclerosis is the common cause of many other cardiovascular diseases, which in total accounts for 17.9 million deaths or 32% of all deaths per year worldwide [1]. For convenience, the pathogenesis of atherosclerosis is divided into three “stages”: disease initiation, progression and advanced (with further complications). Atherosclerosis is a complex pathological process which includes many risk factors such as dyslipidaemia, diabetes mellitus, hyperglycaemia, obesity, hypertension, unbalanced nutrition, sedentariness, stress, environmental factors and others [2]. Lipid accumulation, inflammation and oxidative damage are considered as key processes in atherosclerosis initiation. The disease progression stage is characterised by further plaque development, accumulation of foam cells, calcification and formation of a necrotic core. In the advanced stage, the growing atherosclerotic plaque forms a blood flow-limiting lesion, which is prone to rupture and trigger acute thrombosis of coronary arteries and causes myocardial infarction.

LDL (low-density lipoprotein) particles are spheroidal packets of cholesterol-rich lipids (LDL-C or “bad cholesterol”), coated with ApoB (Apolipoprotein B) protein, surrounded by a shell of phospholipids and unesterified cholesterol, whose function is to transport fats around the body through the blood. Among other groups of lipoproteins (chylomicrons or ULDL (ultra-low-density lipoproteins), VLDL (very-low-density lipoprotein), IDL (intermediate-density lipoprotein), LDL and HDL (high-density lipoprotein), high LDL is considered as the main cause of atherosclerosis and associated with increased risk of development of other CVDs (cardiovascular diseases) [3,4]. The primary role of LDL-C in disease initiation and progression was supported by data from patients with familial hypercholesterolaemia, who developed atherosclerosis prematurely, and from individuals with variations in *PCSK9* (Proprotein Convertase Subtilisin/Kexin Type 9) gene, which is a crucial regulator of plasma cholesterol homeostasis, associated with lifelong low LDL-C levels [5,6].

**Figure 1:**
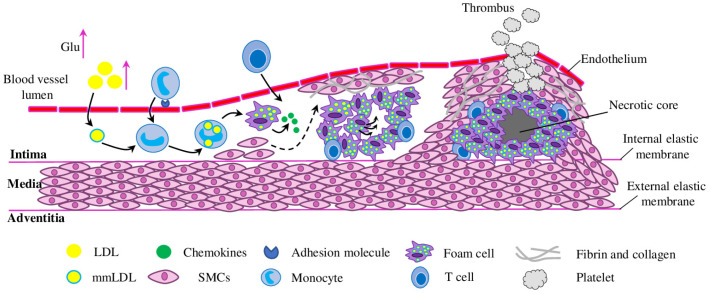
Initiation and progression of atherosclerosis. In the early stage of lesion initiation, different factors (hyperglycaemia and oxidative stress) promote the initiation of atherosclerosis. LDL (low-density lipoprotein) particles accumulate in the intima, where they undergo various modifications that can provide them with pro-inflammatory and immunogenic properties. Activated ECs (endothelial cells) express adhesion molecules which bind monocytes, and chemokines further promote migration of the bound monocytes into the bloodstream. Monocytes maturate into macrophages, bind LDL particles and become foam cells. Less abundant T cells also enter the intima and regulate functions of ECs, SMCs (smooth muscle cells) and innate immune cells. Further migration and proliferation of SMCs towards the injured area generate an atherosclerotic plaque structure, whose growth limits the blood flow and nutrient supply to surrounding tissues. Apoptosis and suppressed efferocytosis inside the lipid core (triple arrows over the foam cell inside the lipid core) lead to secondary inflammation and necrosis, which results in necrotic core formation. Plaque rupture is the common complication of atherosclerosis in advanced stages; it activates thrombotic events, thus completely blocking the blood flow and might cause brain stroke or myocardial infarctions.

Lipid accumulation. The initial stage of lesion formation is characterised by accumulation of LDL particles in the intima, where plasma antioxidants cannot reach, but they can undergo various modifications (desialylation, oxidisation and other modifications)—mmLDL (multiple modified low-density lipoproteins), facilitate foam cell formation, induce inflammation and stimulate humoral and adaptive immunity (Figure 1) [7,8]. Despite significant efforts, available experimental and clinical data could not provide rigorous proof of the causal role of specifically oxidised LDL particles in atherosclerosis initiation [9,10], suggesting, however, other potential targets (such as desialylated LDL) for therapeutic intervention [11]. While the relevance of the exact type (or types) of LDL particle modification to atherosclerosis is an open question, the causative role of LDL-C was indubitably established and confirmed by pharmacological, epidemiological and therapeutic studies (reviewed in [12]).

HDL cholesterol (HDL-C, or “good cholesterol”) levels are often associated with reduced risk of atherosclerotic events; however, the HDL-C increasing therapies did not improve cardiovascular outcomes, thus not supporting the athero-protective role of HDL-C [13]. On the contrary, the causative role of triglyceride-rich lipoproteins and LPA (Lp(A) or Apolipoprotein(A)) in atherosclerosis was supported by substantial human genetic evidence [14,15].

**Figure 2:**
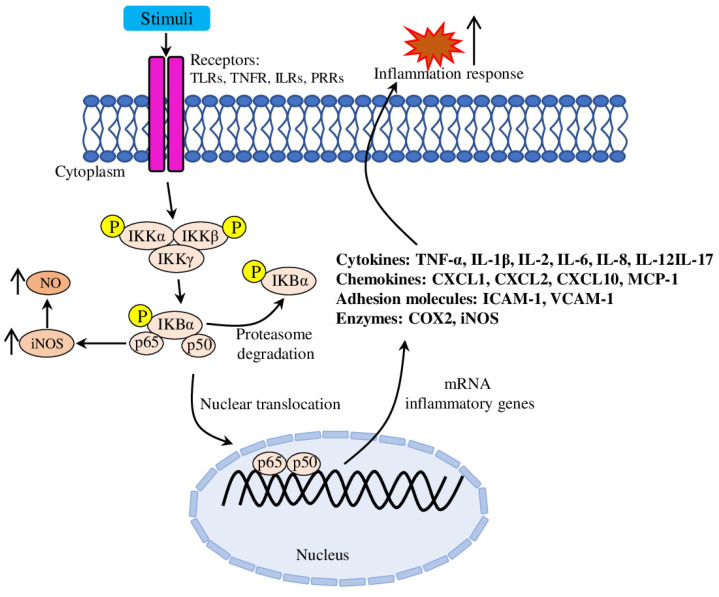
Regulation of the canonical NF-*κ*B (nuclear factor kappaB) pathway. Various stimuli (such as TLR ligands, cytokines and antigens) through a wide variety of signalling adaptors to engage, phosphorylate and degrade the inhibitory protein I*κ*B of the IKK complex. The release from I*κ*B activates NF-*κ*B dimer (p65/p50), allows it (p65/p50) to translocate to the nucleus and promote transcription of the NF-*κ*B-dependent genes (such as cytokines, chemokines, adhesion molecules and others).

Inflammation. Lipid accumulation and the inflammatory immune response are closely linked to atherosclerosis initiation and development. Modified LDLs form aggregates in the blood vessels’ intima, causing EC (endothelial cell) dysfunction, inducing expression of adhesion molecules (such as VCAM-1 (Vascular Cell Adhesion Molecule), ICAM-1 (Intercellular Adhesion Molecule), P- and E-selectins), thus increasing leukocyte adhesiveness (Figure 2). ECs, surrounding smooth muscle cells and recruited leukocytes release various inflammatory mediators, which promote inflammation, stimulate immunity and facilitate further disease progression [16]. Because of the crucial role of inflammation, various inflammatory molecules (such as TNF-*α* (tumour necrosis factor-alpha), IL-1*β* (Interleukin), IL-6, IFN-*γ* (Interferon gamma), CRP (C-reactive protein) and others) serve as important therapeutic targets [17,18], biomarkers of inflammation and cardiovascular risk factors [19].

Furthermore, a rich experimental basis helped to establish a strong link between inflammation and immunity in atherosclerosis development. For example, some subtypes of T cells have an anti-atherogenic role (such as T_reg_ (regulatory T cells)), others pro-atherogenic (such as T_H_1 (T helper 1)), while the role of many other subsets (CD8^+^T, *γδ* T, CD28^null^T, T_H_17, T_H_22 and many others) are much less understood [20]. Similarly, targeting of innate and adaptive immunity responses was effective in modulating experimental atherosclerosis, suggesting its high potential in clinical use [21].

Oxidative damage. In addition to inflammation, the oxidative stress is considered as a key player in the initiation and progression of endothelial dysfunction and atherosclerosis [22]. Dysregulation of the antioxidant system and enhanced production of ROS (reactive oxygen species) promote endothelial injury, exacerbate inflammatory stress and accelerate lipid deposition in the pathogenesis of atherosclerosis [23]. ECs are a layer of cells bordering the bloodstream and vascular smooth muscle and regulating vascular permeability and secretion of bioactive substances, which are further associated with anticoagulant, haemostatic and antithrombotic functions. The endothelial monolayer separates the blood flow and the arterial intima and occurs as the site of plaque (atheroma) initiation during early atherogenesis [24]. Initial vascular remodelling of the innermost vascular layer is driven by lipid accumulation, on-site macrophage recruitment and uptake of mmLDL, which is further transformed into foam cells in the atherosclerotic plaque [25]. Further, release of inflammatory cytokines and pro-apoptotic regulators promotes a process of epithelial-to-mesenchymal transition and fibroblast-to-myofibroblast differentiation, thus reinforcing endothelial dysfunction and progression of the atherosclerotic lesion. In the later stages, the lipid core could necrotise and become covered by a thin and often calcified fibrous cap, which is prone to rupture and could lead to a thrombus and vessel occlusion [26].

### Initiation of Atherosclerosis

The exposure of ECs to atherogenic risk factors interferes with normal production of endogenous vasodilators (specifically, the most important one, NO (nitric oxide)) [27]. The normal vascular blood flow is mechanotransducting, thus effecting EC function and homeostasis via shear and stretch stresses and wall hydrostatic pressure. Among these mechanical effects, shear stress is considered as the most important factor in atherosclerosis development through the activation of eNOS (endothelial NO synthase) via mechanosensitive transcription factor (such as KLF2 (Kruppel-like factor 2)) [28]. However, pathological stimuli reduced eNOS levels in ECs and promoted production of the vasoconstrictor ET-1 (endothelin-1), PAI-1 (plasminogen activator inhibitor-1) and transcription regulator HIF-1 (hypoxia-inducible factor 1), thus increasing platelet aggregation, endothelial procoagulant effect and smooth muscle cell proliferation and migration, which, in total, resulted in increased risk of thrombosis and atherosclerosis [29].

**Figure 3:**
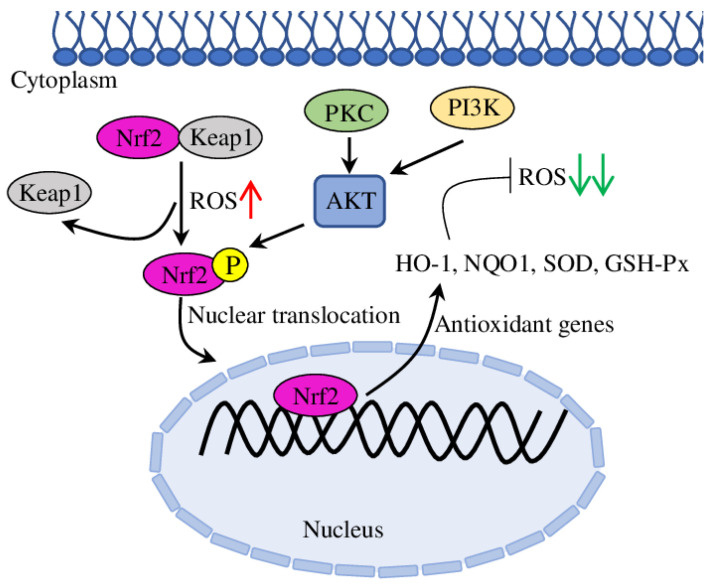
The regulation of Nrf2/HO-1 pathway. The high ROS level releases Nrf2 from Keap1 protein, which allows it to be translocated to the nucleus and to activate antioxidant genes (such as *HO-*1, *SOD* and *NQO1*).

**Figure 4:**
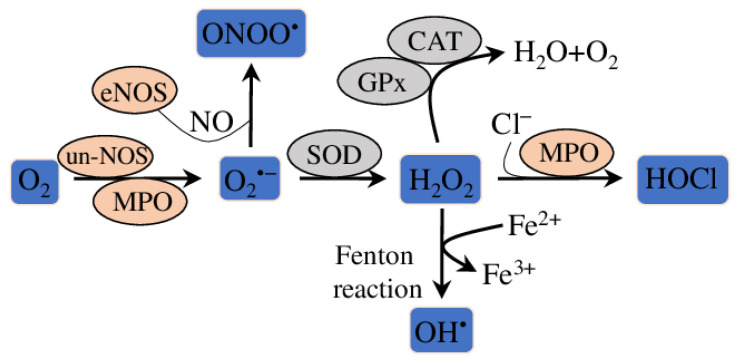
Schematic outline of the interrelationships between reactive oxygen species (ROS) and antioxidative enzymes. O_2_^•−^ (superoxide) is produced from O_2_ by various enzymes (such as MPO (myeloperoxidase), un-eNOS (uncoupled endothelial nitric oxide synthase)) from different metabolic pathways. Without cofactor, eNOS shifts to a monomeric form (uncoupled) and produces superoxide anions instead of synthesising NO. Also, superoxide can react with NO (nitric oxide) to form ONOO^•^. SOD (superoxide dismutases) can convert superoxide to H_2_O_2_ (hydrogen peroxide). In the presence of Fe^2+^ (Fenton reaction) H_2_O_2_ can spontaneously convert to OH^•^ (hydroxyl radical). Various antioxidants (CAT, Gpx) can detoxify H_2_O_2_ to H_2_O and O_2_. MPO (myeloperoxidase) can oxidize chloride with H_2_O_2_ to form the strong-oxidizing agent HOCl (hypochlorous acid).

Nrf2 (Nuclear Factor (Erythroid-Derived 2)-Like 2) is a crucial transcription factor regulating a complex regulatory network, including also oxidative stress response in cells (Figure 3). Nrf2-mediated transcription regulates the expression of downstream antioxidant gene (such as *SOD* (superoxide dismutase), *NQO1* (NAD(P)H: Quinone Oxidoreductase 1), *HO-1* (Heme Oxygenase 1) or *GSH-Px* (glutathione peroxidase)), which remove ROS and protect cells from oxidative stress (Figure 4) [30]. Additionally, other proteins, such as PKC (protein kinase C), KEAP1 (Kelch-like ECH Associated Protein 1), AKT (protein kinase B) and PI3K (Phosphoinositide 3-Kinase Alpha), regulate Nrf2 transcription and induce phosphorylation [31]. Among others, the Nrf2/HO-1 pathway is the best studied for its antioxidative potential to protect ECs from oxidative stress-mediated injury, endothelial dysfunction and, subsequently, atherosclerosis [32,33,34].

Progression of atherosclerosis. As the disease progresses, atherosclerotic plaques continue to accumulate lipid particles and blood cells to give rise to foam cells. Also, metaplasia of smooth muscle cells can contribute to foam cell formation and atherosclerotic plaque growth [35]. Smooth muscle cells and macrophages are subjected to programmed cell death, which, in combination with impaired efferocytosis (dead cells clearance process), leads to the formation of the plaque necrotic core (Figure 1) [36]. Furthermore, dysregulation of calcium homeostasis and impaired clearance in the plaque activates a mineralisation process, similar to bone formation [37]. Such spotty calcification usually increases plaques instability, which promotes a tendency to rupture and provoke thrombosis, while extended calcification can stabilise plaques [38].

Advanced stages and complications of atherosclerosis. Eventually, the growing atherosclerotic plaque starts to invade the arterial lumen, impairing normal blood flow. Such a complication of coronary arterial perfusion can produce symptoms of angina pectoris and ischaemia, especially during physical effort, when the myocardial demand for oxygen increases [39]. “Vulnerable” atherosclerotic plaques are characterised by a large lipid core covered with a thin fibrous cap. Such plaques are more prone to rupture and trigger acute thrombosis of coronary arteries and cause myocardial infarction (Figure 1). “Stable” plaques, on the contrary, have thicker fibrous caps over limited lipid cores [40]. Plaque erosion is an alternative thrombotic mechanism for lesions with a distinct morphology (few inflammatory leukocytes and little lipids in the cores, and a rich extracellular matrix without a thin, friable fibrous cap). In comparison to plaque rupture, the molecular mechanisms of plaque erosion are much less studied [41].

Therefore, beneficial treatment for atherosclerosis usually provides anti-inflammatory, antioxidant and/or lipid-lowering properties. In addition to well-known drugs and substances specifically designed for atherosclerosis treatment [42], many drugs used to treat other diseases (such as diabetes, obesity and different types of cancer) have been repurposed for atherosclerosis. Additionally, many natural bioactive compounds delivered from different plants have proven anti-atherosclerotic activities [28,43].

Essential oils (EOs) are volatile compounds widely presented in different parts of plants (flowers, seeds, roots, leaves and stems) and globally used as food, perfumes, cosmetics and, most importantly, in traditional medicine to treat various diseases [44,45]. EOs are usually divided into three subgroups: acyclic (such as nerol, linalool, geraniol, citronellol, myrcene and others), monocyclic (such as carvone, menthol, *α*-terpineol, terpinolene, limonene, pulegone, phellandrene and others) and bicyclic (such as fenchone, thujone, camphor, *α*-pinene and others). Various technologies have been developed for effective extraction of EOs and treatment of hypertension, diabetes and obesity [46]. The health beneficial effects of EOs are mostly associated with terpenes, which are a broad group of natural substances classified into several groups based on the length of their carbon chains. Among all groups, mono-terpenes have the most significant biomedical activities in aromatic plants [47]. Specifically, the role of plant EOs and some of their isolated constituents, such as monoterpenoids, was demonstrated to improve cardiovascular function and reduce cardiovascular diseases risk factors [48,49]. Further in this review, we focus on the functional and mechanical anti-atherosclerotic role of EOs extracted from various plant sources. In particular, anti-inflammatory, anti-oxidative and lipid-lowering properties of EOs investigated in various in vitro and in vivo systems are discussed. Additionally, some relevant clinical trials evaluating the beneficial properties of EOs and known limitations on EO-based studies are highlighted.

## 2. Anti-Inflammatory Effect of EOs

Inflammation is a part of the innate immune defensive response of the organism against harmful stimuli, such as physical injury, chemicals, pathogens, viruses and others. Inflammation is involved in a variety of pathological processes; its regulation is complex and coordinated by many inflammatory mediators whose surplus production could lead to chronification of the inflammation process and initiate development of various diseases [50]. Inflammation is considered as a major factor implicated in the initiation and progression of atherosclerotic processes [51]. LPS (lipopolysaccharide) is the main component of Gram-negative bacteria, and thus is often used in in vitro experiments to imitate bacterial infection and promote secretion of pro-inflammatory cytokines, nitric oxide and eicosanoids [52]. Mechanically, LPS binds TLR4 (Toll-like receptor 4) and activates both MyoD88-dependent and MyoD88-independent signalling pathways. The MyoD88-dependent pathway activates NF-*κ*B (nuclear factor kappaB) and MAPK/AP-1 (mitogen activated protein kinase/activator protein-1) signalling, and the MyoD88-independent pathway regulates IRF3 (interferon regulatory factor 3), while both MyoD88 pathways trigger pro-inflammatory cytokine transcription [53]. Further in this section, we discuss recent papers elucidating the molecular mechanisms of the anti-inflammatory activities of plant-derived EOs.

### 2.1. In Vitro Raw 264.7 Macrophage Test System

Research over the last decades attempted to identify and characterise EOs with the most pronounced anti-inflammatory effect and to decipher the underlying molecular mechanisms (Table 1). A desert medical and aromatic plant *Artemisia judaica* L., widely used in traditional medicine as an anti-bacterial and anti-fungal agent, was recently characterised by anti-inflammatory activity. As was shown in in vitro culture of LPS-treated macrophages, EO extracted from *A. judaica* inhibits NO production with low toxicity to macrophages. Bioactive components were mostly represented by mono-terpenes with piperitone, camphor and ethyl cinnamate as the main compounds [54]. Similarly, NO-inhibiting activity was determined in the EO derived from the Chinese traditional herb *Waldheimia glabra* (Decne), where α-bisabolol, valeranone and chamazulene were dominating compounds [55]. Anti-inflammatory activity also has been determined in EO derived from *Hibiscus sabdariffa* L., with n-Hexadecanoic acid as the most abundant volatile component. Treatment of LPS-stimulated macrophages with *H. sabdariffa* EO greatly inhibited the activation of JNK (C-Jun N-Terminal Kinase 1) and ERK1/2 (extracellular signal-regulated protein kinase 1 and 2) signalling pathways and decreases *iNOS* (inducible nitric oxide synthase) expression, thus reducing the production of NO and pro-inflammatory cytokines (IL-1, IL-6, TNF-α and COX-2) [56]. EO derived from the blossoms of *Citrus aurantium* L. has shown similar anti-inflammation activities: reduced levels of NO, IL-1β, IL-6, TNF-α and COX-2, and decreased expression levels of their genes. Additionally, *C. aurantium* EO suppressed JNK and p38 phosphorylation, inhibited NF-*κ*B and I*κ*B kinase activation, and I*κ*B*α* phosphorylation and degradation in LPS-treated macrophages. Active components responsible for observed anti-inflammation activity were characterised as linalool, *α*-terpineol and (R)-limonene [57]. Similar properties were shown for related species *L. luisieri* (Rozeira) Rivas Mart. and *L. pedunculata* (Mill.) Cav. with 1,8-cineole and fenchone as prevalent compounds. *L. luisieri* EO effectively impaired nuclear translocation of NF-*κ*B/p65, thus reducing iNOS levels. Interestingly, the expression of pro-*IL-1β* gene was increased, while the expression of *COX-2* was not changed. On the contrary, *L. pedunculata*-derived EO was less effective in NO inhibition. Further investigation suggested that necrodane derivatives (trans-*α*-necrodol and trans-*α*-necrodyl acetate), the minor component of *L. luisieri* EO, are the most potent inhibitors of NO production [58].

Recently, anti-inflammatory properties have been characterised in traditional Chinese herbs *Siegesbeckia pubescens* (Makino) and *Siegesbeckia orientalis* L., whose EOs reduced NO production and IL-6 release in LPS-stimulated macrophages, respectively. The main components of the EO were β-caryophyllene oxide, trans-longipinocarveol and dehydrosaussurea lactone in *S. pubescens* and β-caryophyllene, spathulenol and β-caryophyllene oxide in *S. orientalis* [59]. Similar properties were found in EO derived from *Thymus camphoratus* (Hoffmanns. and Link) and *T. carnosus* (Boiss.), which inhibited NO production and reduced expression of *iNOS* and *COX-2* genes. Borneol and camphene were defined as the active components of *T. carnosus* EO, while *T. camphoratus* EO was rich in borneol and 1,8-cineole [60]. EO from *Santolina rosmarinifolia* L., composed mainly of β-pinene, borneol, myrcene and limonene, decreased NO and pro-IL-1*β* release and expression of *iNOS* in LPS-stimulated macrophages [61]. Interestingly, EO derived from *Cirsium japonicum* (Maxim.) demonstrated anti-inflammatory activity (inhibiting NO production in LPS-treated macrophages) combined with lipid-lowering activity (decreased lipid accumulation in ox-LDL-induced (oxidised) macrophages). However, as the active components were not defined in this particular study, further research is required to elucidate the molecular mechanisms responsible for these activities [62].

Anti-inflammatory properties have been demonstrated in the popular spice oregano (*Origanum vulgare* L.) EO. The results obtained showed that treatment of LPS-treated macrophages with oregano oil effectively inhibited the expression and secretion of IL-1*β*, IL-6 and TNF-*α*. A suggested molecular mechanism of *O. vulgare* oil’s action was defined through the NADPH (nicotinamide adenine dinucleotide phosphate) oxidase pathway [63]. The active components of the *O. vulgare* oil were identified by other researchers as carvacrol, thymol, *γ*-terpinene and *ρ*-cymene [64,65].

**Table 1:** Plant essential oils with potential anti-atherosclerotic effects.

Species	Major Component	Effect	Experiment Setup	References
*Artemisia* *judaica*	piperitone, camphor, ethyl cinnamate	inhibits NO production	LPS treated Raw 264.7 macrophage	[54]
*Hibiscus sabdariffa*	*n*-Hexadecanoic acid	inhibits NO, IL-1, IL-6, TNF-*α* and COX-2 production; JNK and ERK1/2 pathways		[56]
*Waldheimia glabra*	*α*-bisabolol, valeranone and chamazulene	inhibits NO production		[55]
*Citrus* *aurantium*	linalool, *α*-terpineol and (R)-limonene	inhibits NO production; reduces IL-1*β*, IL-6, TNF-*α* and COX-2 protein levels and gene expression; inhibits NF-*κ*B/65 and I*κ*B activation		[57]
*Siegesbeckia pubescens*	β-caryophyllene oxide, trans-longipinocarveol and dehydrosaussurea lactone	inhibits NO production		[59]
*Siegesbeckia orientalis*	β-caryophyllene, spathulenol and β-caryophyllene oxide	inhibits IL-6 release		
*Origanum* *vulgare*	carvacrol, thymol, *γ*-terpinene and *ρ*-cymene	inhibits IL-1*β*, IL-6 and TNF-*α* expression and secretion	LPS treated Raw 264.7 macrophage	[63] [64,65]
*Thymus* *camphoratus*	1,8-cineole and borneol	inhibits NO production; reduces expression of *iNOS* and *COX-2* genes		[60]
*Thymus* *carnosus*	borneol and camphene			
*Santolina* *rosmarinifolia*	*β*-pinene, borneol, myrcene and limonene	decreases NO and pro-IL-1*β* release and expression of *iNOS*		[61]
*Rosa rugosa*	citronellol and geraniol	reduces production of IL-1*β*, IL-6 and TNF-*α*; increases CAT and SOD activities; inhibits MDA section; normalises iNOS, NO and ROS levels		[66]
*Cirsium* *japonicum*	-	inhibit NO production		[62]
		decreases lipid accumulation	ox-LDL-induced Raw 264.7 macrophages	
*Lavandula* *pedunculata*	1,8-cineole and fenchone	inhibits NO production	LPS treated Raw 264.7 macrophage	[58]
*Lavandula luisieri*	1,8-cineole and fenchone; trans-*α*-necrodol and trans-*α*-necrodyl acetate	reduces *iNOS* and increases *pro-IL-1β* expression; impairs nuclear translocation of NF-*κ*B/p65		
	5-Methylene-2,3,4,4- tetramethylcyclopent-2- enone; 1,8-cineole	reduces TNF-*α* and inhibits CCL2 release	LPS-stimulated THP-1 cells	[67]
*Thymbra* *capitata*	carvacrol	reduces TNF-*α* release		
*Cymbopogon commutatus*	geraniol	inhibits IL-1*β*, IL-6 and TNF-*α*; down-regulates ICAM-1 mRNA level; suppresses IkBα phosphorylation; NF-*κ*B/p65 activation and nuclear translocation; increases *HO-1* expression	ox-LDL-induced HUVECs	[68]
*Lavandula* *angustifolia*	linalool, linalyl acetate and terpinen-4-ol	decreases IL-1*β*, IL-6, IL-8 and TNF-*α* mRNA and protein levels	LPS-treated THP-1 macrophages	[69]
	linalool, linalyl acetate	suppresses TNF-*α*-induced expression of *E-selectin*, *P-selectin*, *VCAM-1*, *ICAM-1* and phosphorylated NF-*κ*B/p65 in the nucleus translocation; inhibits TNF-*α*-induced increase in E-selectin mRNA levels in HUVECs	TNF-α-stimulated bEnd.3 and HUVEC	[70]
*Rosmarinus* *officinalis*	1,8-cineole, camphor, limonene and *α*-pinene	reduces total cholesterol, LDL, triglycerides, abdominal fat gain	triton and coconut fat-induced rat model of dyslipidaemia	[71]
	*α*-pinene, camphor, and 1,8cineole	increases IL-10 level	LPS treated THP-1 macrophage	[72] [73]
	camphor, borneol, *α*-pinene, 1,8-cineole	reduces glucose, triglyceride, total cholesterol; increases HDL; improves serum enzymes (AST, ALT and ALP); ameliorates lipid deposition, macrophage infiltration and stenosis of hepatic vein	rats on HFD	[74]
*Zingiber* *officinale*	1,8-cineole, terpineol, linalool, borneol			
*Monarda* *didyma*	carvacrol, *p*-cymene and thymol	decreases IL-6 and increases *miR-146a* expression	LPS treated U937 lymphoma cell line	[75]
*Platycladus orientalis*	-	increases IL-10 and reduces IL-1*β* and TNF-*α* content in the serum; reduces the p65 and I*κ*B phosphorylation level	mice and rats’ models	[76]
*Salvia* *officinalis*	1,8-Cineole, *α*-caryophyllene	reduces body weight gain, liver and kidney weight; prevents lipid accumulation and focal necrosis in the liver; attenuates haemorrhage foci, reduces Bowman’s space and necrotic epithelial cells lining the tubules in the kidney; normalises antioxidant enzymes (SOD, CAT and Gpx) activity, the lipid profile (total cholesterol, triglyceride, LDL, VLDL and total lipids) and blood biochemical parameters (GGT, pancreatic lipase, AST, ALT, LDH, ALP and CPK; reduces the levels of TBARS and protein carbonyls; increases cholesterol and triglyceride faecal excretion	HFD mice model	[77]
*Psidium* *guineense*	spathulenol	in vitro antioxidant activities (MDA, ABTS and DPPH); reduces inflammation in carrageenan-induced paw oedema and pleurisy models	in vitro test systems and mice	[78]
*Alpinia* *zerumbet*	*β*-pinene, *α*-cadinol and camphor	decreases *ICAM-1* and *VCAM-1* expression, NF-*κ*B/p65 phosphorylation and nuclear translocation, IKK*α*/*β* phosphorylation and increases I*κ*B*α* protein levels; reduces lactate dehydrogenase release and caspase-3 activation	LPS-induced HAEC and mice	[79] [80]
		inhibits p65 subunit nuclear translocation, secretion of IL-8, TNF-*α*, ICAM-1 and VCAM-1	high-glucose treated HUVEC	[81] [80]
*Eucalyptus globulus*	1,8-cineole	reduces levels of TNF-*α*, NO, IL-1*α* and IL-1*β*; reduces phosphorylation of NF-*κ*B/p65 and p38, and increases of ERK1/2 and JNK1/2; down-regulates TREM-1, NLRP3 and MKP-1	LPS-treated alveolar macrophage cell line MH-S	[82]
-	1,8-cineole (purified)	reduces levels of IL-1 and IL-6; reduces phosphorylation of JNK1/2, and increases of NF-*κ*B/p65; down-regulates MKP-1		
-	1,8-cineole (purified)	reduces IL-6 and IL-8 secretion; normalises NO levels; improves *iNOS* expression and eNOS protein levels; decreases p65 phosphorylation and nuclear translocation	LPS-induced HUVEC	[83]
-	1,8-cineole and *α*-pinene (purified)	decreases TNF-*α*, IL-1*β*, IL-6 and eNOS mRNA levels		[84]
-	1,8-cineole (purified)	inhibits IL-1*β*, IL-6 and IL-8, and promotes IL-10 release; reduces NF-*κ*B/p65 and VCAM-1, and increases PPAR-γ expression	LPS-treated mice	[85]
-	bornyl acetate	reduces *E-selectin*, *ICAM-1* and *VCAM-1*, *IL-1β* and *TNF-α* expression; ameliorates reduction in cell viability	ox-LDL-induced HUVECs	[86]
-	β-Elemene	reduces aortic root lesion sizes and necrotic core areas; increases the plaque stability score; increases expression of *eNOS*, *CAT*, *Gpx* and *GSH*; reduces levels of p22phox, IL-1*β*, TNF-*α*, INF-*γ*, MCP-1 and ICAM-1	ApoE^−/−^ mice	[87]
-	citronellal	reduces atherosclerotic plaque size; alleviates arterial stenosis; reduces production of IL-1, IL-6, IACM-1 and VACM-1; normalises levels of NO, MDA and SOD activity	rats on HFD	[88]
-	β-caryophyllene	inhibits VCAM-1; reduces total cholesterol and triglycerides in serum	TNF-*α*-stimulated HUVECs and mice	[89]
-	geraniol	increases Gpx, GST, mtSOD, GSH and NQO1; reduces TBARS; enhances *Nrf2* and *HO-1* expression; decreases IL-1*β*, IL-18 and TNF-*α* levels, NF-*κ*B/65 activation and nuclear translocation	doxorubicin-treated rats	[90]

HUVEC—human umbilical vein endothelial cell; HAEC—human aortic endothelial cells; HFD—high fat diet.

### 2.2. Other In Vitro Test Systems

Anti-inflammatory properties have also been described in several lavender species. Thus, EO extracted from *Lavandula angustifolia* Mill., with linalool, linalyl acetate and terpinen-4-ol as the main bioactive components, inhibited the synthesis of four pro-inflammatory cytokines (IL-1*β*, IL-6, IL-8 and TNF-*α*) in LPS-stimulated THP-1 cells [69]. Another study has also supported the effect of *L. luisieri* EO on LPS-stimulated THP-1 cells by reducing TNF-*α* and inhibiting CCL2 (C-C motif chemokine ligand 2, or MCP-1 (Monocyte Chemoattractant Protein-1)) release. Accordingly, *Thymbra capitata* (L.) Hoffmanns. and Link) EO was effective only in reducing TNF-α release. The main components for *L. luisieri* and *T. capitata* were 5-Methylene-2,3,4,4-tetramethylcyclopent-2-enone, 1,8-cineole and carvacrol, respectively [67]. Similarly, the EO derived from *Rosmarinus officinalis* (Spenn.) and tested in a LPS-treated THP-1 macrophage model, increased levels of the anti-inflammatory IL-10, while the levels of pro-inflammatory cytokines IL-6 and TNF-*α* were not changed [72]. The active components of the *R. officinalis* EO were defined as α-pinene, camphor and 1,8-cineole in another research [73].

Interesting results were obtained with the application of *Eucalyptus globulus* (Labill.) EO on LPS-induced murine lung alveolar macrophage cells. The levels of pro-inflammatory mediators (TNF-*α*, NO, IL-1*α* and IL-1*β*) were reduced by *E. globulus* EO treatment, while the purified major component 1,8-cineole reduced only levels of IL-1 and IL-6. Also, the expression of *TREM-1* (Triggering Receptor Expressed On Myeloid Cells 1) and PRR (pattern recognition receptor) *NLRP3* (NLR Family Pyrin Domain Containing 3) of the inflammasome were downregulated by *E. globulus* EO treatment. Interestingly, the phosphorylation of NF-*κ*B/p65 and p38 was attenuated, while ERK1/2 and JNK1/2 increased. On the contrary, the effect of 1,8-cineole on NF-*κ*B and JNK1/2 was opposite, while both (*E. globulus* EO and pure 1,8-cineole) treatments downregulated *MKP-1* (Dual Specificity Phosphatase 1) phosphatase, a negative regulator of MAPKs [82]. The EO extracted from the flowering parts of *Monarda didyma* L. have affected Toll-like receptor-4 signalling pathway by decreasing IL-6 and increasing *miR-146a* expression in LPS-treated U937 cells (a pro-monocytic, human histiocytic lymphoma cell line). Among 20 defined bioactive compounds, carvacrol, *p*-cymene and thymol were the most abundant [75].

Pure 1,8-cineole was demonstrated to ameliorate dysfunction of LPS-induced HUVEC (human umbilical vein endothelial cells). The defined molecular mechanism suggested that 1,8-cineole reduced IL-6 and IL-8 secretion and normalised NO levels. Also, *iNOS* expression and eNOS protein levels were improved, alongside decreased p65 phosphorylation and nuclear translocation [83]. Similarly, application of pure 1,8-cineole and *α*-pinene decreased TNF-*α*, IL-1*β*, IL-6 and eNOS mRNA levels in LPS-induced HUVEC cells [84].

### 2.3. EO Effect on Adhesion Molecules and Leukocytes Recruitment In Vitro and In Vivo

The recruitment of the leukocytes to the sight of inflammation is regulated by the NF-*κ*B transcription factor and usually include such cell adhesion molecules as E-selectin, P-selectin, VCAM-1 (vascular cell adhesion molecule-1) and ICAM-1 (intercellular adhesion molecule-1) [91]. Recently, EO extracted from a dry fruit from *Alpinia zerumbet* (Pers.) B.L.Burtt and R.M.Sm. decreased expression of *ICAM-1* and *VCAM-1* and NF-*κ*B p65 phosphorylation and nuclear translocation in both LPS-induced HAEC (human aortic endothelial cells) and in vivo mice models. Also, LPS-mediated increase in IKK*α*/*β* phosphorylation and reduction in I*κ*B*α* protein levels were prevented in HAEC by *A. zerumbet* oil treatment. Interestingly, *A. zerumbet* oil demonstrated also an anti-apoptotic effect on LPS-induced HAEC cells, which was evaluated by reduced lactate dehydrogenase release and caspase-3 activation. While the active components of the *A. zerumbet* oil were not defined in this work [79], other research suggested that *β*-pinene, *α*-cadinol and camphor were the major components responsible for the anti-inflammatory effect [80]. These results were confirmed by another study, where HUVECs injury was achieved with high glucose treatment. *A. zerumbet* oil treatment inhibited cytokines (IL-8, TNF-*α*) and adhesion molecules (ICAM-1 and VCAM-1) secretion and p65 subunit nuclear translocation, inhibiting NF-*κ*B signalling pathway activation and reducing HUVEC injury [81].

The effect of *Lavandula angustifolia* EO and its main components (pure solutions of linalyl acetate and linalool) on the expression of adhesion molecules was explored in TNF-*α*-stimulated murine brain endothelial bEnd.3 cells and HUVECs. Native *L. angustifolia* EO and pure linalyl acetate suppressed TNF-*α*-induced *E-selectin*, *P-selectin*, *VCAM-1*, *ICAM-1* expression and phosphorylated-NF-*κ*B p65 translocation to the nucleus in bEnd.3 cells, while linalool had no effect on *P-selectin* or phosphorylated-NF-*κ*B p65 translocation. Furthermore, in HUVEC native *L. angustifolia* EO inhibited TNF-*α*-induced increase in E-selectin mRNA levels [70].

Geraniol, an active component of lemongrass (*Cymbopogon commutatus* (Steud.) Stapf.) EO, was characterised as an effective anti-inflammatory agent. In the experimental system of ox-LDL-induced HUVECs inflammation, application of geraniol inhibited production of pro-inflammatory cytokines (IL-1*β*, IL-6 and TNF-*α*), down-regulated ICAM-1 mRNA level, increased *HO-1* expression and suppressed activation and nuclear translocation of NF-*κ*B p65 and phosphorylation of I*κ*B*α* [68]. In addition to native EOs, extracted from different plants, the effect on adhesion molecules and leukocyte recruitment was shown also for their purified components. For example, bornyl acetate reduced the expression of *E-selectin*, *ICAM-1* and *VCAM-1* in ox-LDL-treated HUVECs, thus inhibiting THP-1 monocyte attachment. Also, bornyl acetate reduced expression of the pro-inflammatory cytokines *IL-1β* and *TNF-α*, and ameliorated reduction in cell viability of ox-LDL-treated HUVECs [86].

Similarly, target delivery of 1,8-cineole with a self-microemulsifying system ameliorated LPS-induced endothelial injury in mice by inhibiting pro-inflammatory cytokines (IL-1*β*, IL-6 and IL-8) and promoting a simultaneous increase in anti-inflammatory IL-10 cytokine release. Also, the expression levels of NF-*κ*B *p65* and *VCAM-1* were reduced, while levels of *PPAR-γ* (peroxisome proliferator-activated receptor *γ*) (NF-*κ*B activation suppressor) was increased [85]. The anti-inflammatory effect of EO from *Platycladus orientalis* L. leaves was investigated on rat and mouse models in vivo. As a result of EO application, paw and granuloma swellings in rats were reduced, and intraperitoneal capillary permeability and auricular swelling in mice were inhibited. Also, the pathological damage of lung tissue was alleviated. Mechanically, EO treatment increased IL-10 and reduced IL-1*β* and TNF-*α* content in the serum and reduced the p65 and I*κ*B phosphorylation level in the NF-*κ*B pathway. However, the active components of the *Platycladus orientalis* EO were not defined in this research [76]. Finally, pure β-caryophyllene effectively inhibited VCAM-1 induction through the JAK2/STAT1/IRF-1 pathway in both in vitro and in vivo model systems (TNF-*α*-stimulated HUVECs and mice, respectively). In particular, β-caryophyllene is an agonist of CB2R (cannabinoid receptor 2), which, among many other genes, regulates *IRF-1* and *VCAM-1* expression. Additionally, β-caryophyllene prevented attachment of THP-1 macrophages to HUVECs and reduced total levels of cholesterol and triglycerides in serum [89].

In total, the results of multiple in vitro and in vivo studies suggested that the NF-*κ*B pathway is the major target for EO-mediated anti-inflammatory effects (Table 1). The disruption of the normal NF-*κ*B pathway functioning (subunits phosphorylation and nuclear translocation) is the primary event responsible for subsequent reduction in levels of pro-inflammatory cytokines, chemokines and adhesion molecules.

## 3. Anti-Oxidative Effect of EOs

ROS and alterations of antioxidant system are crucial triggers in initiation and progression of endothelial dysfunction and atherosclerosis. Recently, the anti-atherosclerotic activities of the *β*-elemene (the main component of the *Curcuma Wenyujin* (Y.H. Chen and C. Ling) EO), have been investigated in vivo in ApoE^−/−^ mice. *β*-elemene effectively reduced the aortic root lesion sizes and necrotic core areas, increased the plaque stability score and increased *eNOS* expression. Also, the expression of antioxidant enzymes (*CAT* (catalase), *Gpx* (glutathione peroxidase) and *GSH* (Glutathione)) in the aorta was up-regulated by *β*-elemene, while the protein level of p22phox (a ROS-producing enzyme) was decreased. Furthermore, the levels of cytokines (IL-1*β*, TNF-*α* and INF-*γ*) and adhesion molecules (MCP-1 and ICAM-1) in the arteries were reduced by *β*-elemene treatment [87]. These data suggest that *β*-elemene combines both anti-inflammation and anti-oxidative stress activities, thus making it an effective anti-atherosclerotic natural agent.

Recently, combined anti-oxidative and anti-inflammatory properties were shown for EO extracted from *Psidium guineense* Sw. leaves, with spathulenol as a major bioactive component. Application of both (*P. guineense* EO and spathulenol) agents demonstrated high antioxidant activities in in vitro systems (MDA (Malondialdehyde), ABTS (ethylbenzothiazoline-6-sulfonic acid) and DPPH (2,2-diphenyl-1-picrylhydrazyl)), while anti-inflammatory properties were demonstrated with oral administration in vivo in mice carrageenan-induced paw oedema and pleurisy models [78]. Similarly, the EO of *Rosa rugosa* Thunb., where citronellol and geraniol were major bioactive components, demonstrated anti-inflammatory and anti-oxidative activities in LPS-treated RAW 264.7 macrophages, which were evaluated through reduced cytokines production (IL-1*β*, IL-6 and TNF-*α*), increased CAT and SOD activities, inhibited MDA section and normalised iNOS, NO and ROS levels [66].

Furthermore, diverse anti-atherosclerotic activities of pure citronellal were studied in vivo in a rat model with HFD-induced atherosclerosis. Citronellal administration effectively reduced the size of atherosclerotic plaque in carotid arteries and alleviated the stenosis of carotid and retinal arteries in atherosclerotic rats, while the levels of total cholesterol, triglycerides, LDL, HDL and blood glucose were not affected. The anti-inflammatory effect of citronellal was mediated via reduced production of cytokines (IL-1 and IL-6) and adhesion molecules (IACM-1 and VACM-1) in blood, and the anti-oxidative effect via normalised levels of NO, MDA and SOD activity [88]. Similarly, geraniol, isolated and purified from lemon grass, demonstrated beneficial cardioprotective properties in the doxorubicin-induced cardiac damage rat model. In particular, orally delivered geraniol alleviated the state of mitochondrial oxidative stress (via increased Gpx, GST, mtSOD, GSH and NQO1 (NAD(P)H: quinone oxidoreductase 1)), mitochondrial lipid peroxidation (via reduced TBARS (thiobarbituric acid reactive substance)) and enhanced *Nrf2* and *HO-1* expression. Furthermore, application of geraniol reduced doxorubicin-induced inflammation by decreasing levels of cytokines (IL-1*β*, IL-18 and TNF-*α*) and NF-*κ*B p65 activation and nuclear translocation [90].

To sum up, in addition to anti-inflammatory activities, plant-derived EOs demonstrate anti-oxidative properties in both in vitro and in vivo conditions (Table 1). The Nrf2 pathway is the major ROS-activated system, responsible for up-regulated expression and production of various antioxidants (GST, SOD, CAT, NQO1 and Gpx), which reduces oxidative stress and damage to ECs and provides cardioprotective effect.

## 4. Lipid-Lowering Properties of EOs

Dyslipidaemia, an alteration of the lipid metabolism, usually manifested by increased LDL, cholesterol and triglycerides, and decreased HDL, is the major risk factor for the initiation and development of non-alcoholic fatty liver disease, diabetes, metabolic syndrome, atherosclerosis and other cardiovascular diseases [92]. Multiple research evidence suggested that plant-derived EOs could be beneficial for lipid metabolism and alleviate hypercholesterolaemia-associated damage. For example, EOs from rosemary and ginger (*Zingiber officinale* Rosc.) effectively reduced levels of glucose, triglyceride and total cholesterol, and increased levels of HDL and improved serum enzymes (AST (aspartate aminotransferase), ALT (alanine aminotransferase) and ALP (alkaline phosphatase)) in rats on HFD. Also, application of the mixture of oils ameliorates HFD-induced lipid deposition, macrophage infiltration and stenosis of the hepatic vein. The major bioactive components of the rosemary and ginger oils were camphor, borneol, *α*-pinene, 1,8-cineole and eucalyptol, terpineol, linalool, borneol, respectively [74]. Similarly, a wide beneficial effect of avocado (*Persea americana* Mill.) oil on metabolism was demonstrated using an NMR-based urinary metabolomics approach on HFD-fed rats. The main oil components (*β*-sitosterol, Δ5-avenasterol, stigmasterol and campesterol) greatly affect lipid, energy, amino acid and gut microbiota metabolism, thus ameliorating hypercholesterolaemia-induced damage [93].

In addition to the earlier described hypocholesterolaemic effect of rosemary oil, another recent study has confirmed and expand these results. Recent experiments on rats with Triton and coconut fat-induced dyslipidaemia demonstrated anti-dyslipidaemic and anti-atherogenic activities of both native rosemary oil and applied in the form of nanoemulsion. In particular, both oil forms reduced total cholesterol, LDL, triglycerides and abdominal fat gain. Also, EO-treated rats were characterised with absence of atherogenesis in the vascular endothelium. The oil composition was similar to results reported in other papers and included 1,8-cineole, camphor, limonene and *α*-pinene [71].

Recently, the effect of *Salvia officinalis* L. EO on HFD-fed mice was characterised in detail. The main active components were defined as eucalyptol and *α*-caryophyllene. Thus, application of *S. officinalis* oil reduced entire body weight gain and the weight of the liver and kidneys, prevented lipid accumulation and focal necrosis in the liver, attenuated haemorrhage foci and reduced Bowman’s space and necrotic epithelial cells lining the tubules in the kidney. Furthermore, the activity of antioxidant enzymes (SOD, CAT and Gpx), the lipid profile (total cholesterol, triglyceride, LDL, VLDL and total lipids) and blood biochemical parameters (GGT gamma-glutamyltranspeptidase), pancreatic lipase, AST, ALT, LDH (lactate dehydrogenase), ALP and CPK (creatine phosphokinase)) were normalised and the levels of TBARS and protein carbonyls were reduced. At the same time, the levels of faecal excretion of cholesterol and triglyceride were significantly increased, therefore suggesting *S. officinalis* EO as an effective anti-hyperlipidaemic and anti-oxidative agent, with additional hepatoprotective and nephroprotective effects [77].

In total, the beneficial effect of EO on lipid profile, glucose level and blood biochemical parameters was confirmed in several recent studies (Table 1). These data suggest that the described EOs are promising therapeutic agents for the treatment of atherosclerosis and other dyslipidaemia-, hypercholesterolaemia- and hyperglycaemia-associated diseases such as diabetes, metabolic syndrome and obesity. However, the molecular mechanism of these important therapeutic effects is not known and further research is required to elucidate it.

## 5. Clinical Trials Confirming Anti-Inflammatory and Anti-Oxidative Properties of EOs

Essential oils, similar to other plant extracts, have attracted wide interest as sources of natural medicinal products. They have been proven to exert diverse bioactive properties (anti-inflammatory, anti-apoptotic, antioxidant, antibacterial, antiviral and antifungal), even if the molecular mechanism of action has not been completely elucidated. To the best of our knowledge, there were no (or currently ongoing) clinical trials to evaluate the anti-atherosclerotic properties of EOs. However, there were several relevant trials investigating anti-inflammatory and anti-oxidative properties of EOs for treatment of oral diseases and oral care (gingivitis, dental plaque and gingival bleeding) and metabolic syndrome.

The pilot examination of the anti-bacterial properties of commercially available EO-based alcohol-free mouthwash to improve oral hygiene in seniors was not successful [94]. A later randomised controlled clinical trial investigated the anti-inflammatory potential of sage (*Salvia officinalis*) EO-containing mouthwash in elderly subjects. Similarly, the inflammatory signs and evaluated parameters (Sulcus Bleeding Index, Plaque Index, tooth staining, xerostomia and degree of stomatitis) were not significantly different from the placebo group [95]. However, recently, a randomized controlled trial comparing the efficiency of EO-containing mouth rinse and flossing efficacy on dental plaque accumulation and gingivitis in interproximal areas has reported some positive outcomes. While after two weeks of treatment there were no significant differences in case of interproximal gingival inflammation and bleeding, the EO mouth rinse was more effective in reducing interproximal dental plaque accumulation in comparison to dental flossing [96]. Similarly, application of *Carica papaya* L. leaf extract and an alcohol-based EO mouthwash (eucaliptol, menthol, methyl salicylate and thymol) effectively reduced gingival bleeding [97].

Further investigation of long-term application of EO-containing mouthwash (eucaliptol, menthol, methyl salicylate and thymol) confirmed reduction in gingivitis and plaque cases for both alcohol-based and alcohol-free EO-containing mouthwash in comparison to standard mechanical oral hygiene procedures [98]. Another EO-based mouthwash composition (menthol, thymol and eucalyptus) effectively reduced the gingival index, bleeding index and oral hygiene index as a stand-alone treatment and in combination with other substances (such as chlorhexidine, hydrogen peroxide and prebiotic) [99]. Most importantly, a recent randomised controlled clinical trial with diabetes patients reported a significant reduction in the plaque and gingival indices and lower levels of bacteria and volatile sulphur compounds after three months’ use of commercially available EO-based rinse [100].

Oxidative stress and inflammation significantly contribute to the pathogenesis of MetS (metabolic syndrome)—a cluster of interconnected serious disorders (such as dyslipidaemia, hyperglycaemia, central obesity and hypertension), which increase risk of diabetes, atherosclerosis and other cardiovascular diseases [101]. As it was defined in a recent randomized clinical trial, *Cuminum cyminum* L. EO effectively increased SOD, TAC (total antioxidant capacity) and decreased MDA in patients with MetS. *C. cyminum* EO, composed of *γ*-terpinene, *β*-pinene, cuminaldehyde and *p*-cymene, was received by patients in form of a soft gel for 8 weeks [102]. However, further investigation showed that important anthropometric and biochemical parameters (such as body weight, glycaemic status, insulin levels, lipid profile and others) were not affected by *C. cyminum* EO supplementation, except for DBP (diastolic blood pressure), which was significantly lower in the EO-treated group [103].

In sum, despite the fact that the anti-atherosclerotic properties of the EO-based supplementations were not directly confirmed in any type of clinical trials, existing evidence suggested their high potential. In particular, anti-inflammatory and anti-oxidative properties were demonstrated in oral diseases and metabolic syndrome. Likely, to fully reveal anti-atherosclerotic potential and affect biochemical blood parameters, a longer intervention time and different EO doses are required. However, while currently EOs are counted as herbal-based nutraceuticals and their safety, quality and efficacy are less strictly regulated than standard pharmaceuticals, many poor-quality products may be present on the market; therefore, these products might be ineffective.

## 6. Conclusions

Many studies highlight the important role of plant-derived EOs and some of their isolated constituents in the improvement of cardiovascular diseases. Some EOs have anti-inflammatory, anti-oxidative and/or lipid-lowering properties, which are able to promote and protect the cardiovascular system, and regress the atherosclerosis process. EOs and secondary metabolites derived from leaves, flowers, seeds, fruits and other plant organs demonstrate an anti-inflammatory effect on the NF-*κ*B signalling pathway and can ameliorate oxidative stress in the Nrf2/HO-1 signalling pathway. Additionally, some EOs showed the ability to normalise blood lipid profile, glucose and biochemical blood parameters, thus suggesting promising anti-obesity and anti-diabetic properties. Thus, plant-derived EOs favourably interact with three main atherosclerosis risk factors and can contribute to the prevention and treatment of atherosclerosis.

However, the main EO compounds responsible for anti-atherosclerosis effect are not well understood. Natural EOs are multicomponent system, and all components could be successfully identified and purified. However, the biological effect of the native EO application often differs significantly from the application of one purified component, thus suggesting that minor components are crucial for EO activities. Currently, the bioactive properties have been demonstrated only for some of the most abundant components (such as carvacrol, eucalyptol (1,8-cineole), geraniol, thymol and some others), while the majority of EO components are still uncharacterised. Also, taking into account the extremely low abundance of these components, it would require significant efforts to purify them from natural plant sources. Another point to consider is the availability of plant materials (flowers, for example), which could be especially pressing in case of endemic or rare plant species.

Another limitation for EO application is the absence of data regarding their dosage, toxicity, kinetics and duration of use. The majority of recent research was conducted in vitro, on various macrophages, ECs and cancer cell lines, while experiments in vivo are scarce. Therefore, nontoxic concentrations defined for a specific EO or a composition of EOs on a particular cell line cannot be extrapolated to an in vivo model and have to be defined again. Also, EOs are volatile compounds which are expected to enter the organisms through the lungs. Thus, theoretically, EOs should be delivered through inhalation therapy via an ultrasonic nebuliser. Another method of EO delivery is via skin penetration. However, in both these delivery routes (inhalation and skin penetration), the control of the daily dose is too complicated, which leaves the oral administration method as the most reliable.

In the recent clinical trials evaluating anti-inflammatory properties of EO-based oral care products, some promising outcomes were achieved with external application of the active agent (mouth wash). Very limited in vivo data suggested the efficiency of nanoemulsion or soft gel delivery systems to obtain a therapeutic effect on the organism-wide level. However, the detailed investigation of EO-mediated anti-atherosclerotic effects in in vivo model systems and in patients is a substantial but necessary challenge for future studies.

## Data Availability

Not applicable.
